# In situ characterization of mitochondrial Hsp60-Hsp10 chaperone complex under folding stress

**DOI:** 10.1126/sciadv.adw6064

**Published:** 2025-10-22

**Authors:** Mingyu Jung, Minjung Kim, Su Jin Ham, Jongkyeong Chung, Soung-Hun Roh

**Affiliations:** ^1^ School of Biological Sciences, Seoul National University, Seoul 08826, Republic of Korea.; ^2^ Institute of Molecular Biology and Genetics, Seoul National University, Seoul 08826, Republic of Korea.

## Abstract

Mitochondrial proteostasis is critical for maintaining mitochondrial function, and its disruption induces mitochondrial unfolded protein response, which up-regulates chaperones to alleviate protein-folding stress. However, how these chaperones mitigate protein-folding stress remains unclear. Here, using correlated cryo–electron tomography, we show that folding stress triggers marked mitochondrial morphological changes, including the accumulation of amorphous protein aggregates and increased abundance and spatial clustering of the mitochondrial heat shock protein 60-heat shock protein 10 (mtHsp60-Hsp10) complex. Subtomogram analysis revealed the in situ architecture and conformational heterogeneity of mtHsp60-Hsp10 under stress, which retains its canonical double-ring structure while adopting distinct football, half-football, and bullet-like states. Notably, the mtHsp60-Hsp10 complex encapsulates unstructured substrates through conserved hydrophobic interactions. We further demonstrate that knockdown of the mtHsp60-Hsp10 complex exacerbates folding stress, as evidenced by elevated cellular stress responses and activation of mitophagy. Our study defines the in situ structural properties of the mtHsp60-Hsp10 complex and provides mechanistic insight into how it safeguards mitochondrial proteostasis under folding stress.

## Teaser

The mitochondrial Hsp60-Hsp10 complex clusters and shifts conformation in response to protein-folding stress.

## INTRODUCTION

Mitochondria act as various roles in eukaryotic cells such as energy generation, heat generation, and apoptosis (*1*). The proteostasis in the mitochondria is crucial for maintaining their function. Previous research showed that insoluble fractions of mitochondria and mitochondrial reactive oxygen species (mtROS) were increased under folding stress (*2*, *3*). This leads to impaired translocation of proteins into mitochondria, resulting in the accumulation of mitochondrial precursor proteins in the cytosol (*4*). In response to accumulation of mitochondrial precursor and mtROS in cytosols, heat shock factor 1 is activated and induces the expression of mitochondrial chaperones, including *HSPD1*, *HSPE1*, and *HSPA9* (*2*–*4*). These chaperones were up-regulated at approximately twice the level under the normal conditions and helped maintain mitochondrial proteostasis (*2*, *4*). This signaling pathway is known as the mitochondrial unfolded protein response (mtUPR), which makes cells effectively mitigate folding stress (*4*, *5*). Given its critical role in cellular health, the mtUPR pathway is implicated in a variety of diseases including cancers (*6*), heart failure (*7*), and neurodegeneration (*8*).

Mitochondrial heat shock protein 60 (mtHsp60; gene name: *HSPD1*) is one of the proteins whose expression level is notably increased through mtUPR (*2*, *4*) yet it is the most abundant protein under basal condition within mitochondria (*9*). Functionally, as a group I chaperonin in mitochondria, mtHsp60 forms a complex with the cochaperonin mtHsp10 (gene name: *HSPE1*) and serves as folding machinery by facilitating the proper folding of unfolded proteins (*10*, *11*). In addition, because mtHsp60-Hsp10 complex is the eukaryotic homolog of bacterial chaperonin, GroEL:ES, it has similar biochemical properties with GroEL:ES directing the substrate folding process using their well conserved hydrophobic residues (*10*–*12*). Because of its abundance and functional role, the mtHsp60-Hsp10 complex is considered as a crucial factor to mitigate folding stress (*13*).

Despite their importance, the physiological role of the mtHsp60-Hsp10 complex under folding stress remains unclear. Because mitochondria have small volumes and mtHsp60-Hsp10 complexes are highly abundant, conventional optical methods face challenges in visualizing the spatial organization of mtHsp60-Hsp10 complexes due to the diffraction limit (*14*). This hindered our understanding of the precise mechanisms underlying the role of the mtHsp60-Hsp10 complex under folding stress. In addition to spatial information, regarding the biochemical properties, previous in vitro studies of mtHsp60 and GroEL usually used chemically denatured substrates and saturated nucleotides or nucleotide analogs to characterize biochemical properties (*10*, *15*, *16*). Because the known biochemical properties were acquired from artificial environments, these may not accurately represent the in vivo biochemical properties of the mtHsp60-Hsp10 complex. Thus, the biochemical characterization of the mtHsp60-Hsp10 complex under physiological condition is necessary to fully understand how the mtHsp60-Hsp10 complex maintains the proteostasis of mitochondria. Collectively, these highlight the need for high-resolution approaches to investigating the spatial organization and in vivo biochemical properties of the mtHsp60-Hsp10 complex to understand the mechanism mitigating folding stress.

In this study, we aimed to elucidate molecular organizations within mitochondria under folding stress using in situ cryo–electron tomography (cryo-ET) by treating gamitrinib-triphenylphosphonium (GTPP) to HeLa cells, which causes mitochondrial folding stress via inhibition of mtHsp90 and subsequently triggers mtUPR (*2*, *4*, *17*). We successfully visualized amorphous aggregates, and the mtHsp60-Hsp10 complex exhibited significant increase in clustering and shift of conformational ensembles. In addition, subtomogram averaging of mtHsp60-Hsp10 complex revealed asymmetric interactions between rings and occupancy of nucleotides, which is different from in vitro structural study. Notably, we identified distinct contact sites with substrates via their conserved hydrophobic residues in situ. Furthermore, we observed increased cellular stress following the knockdown of the mtHsp60-Hsp10 complex, confirming that its spatial and structural properties are crucial for mitigating folding stress.

## RESULTS

### Cellular cryo-ET revealed amorphous aggregates within mitochondria under folding stress

**Fig. 1.**
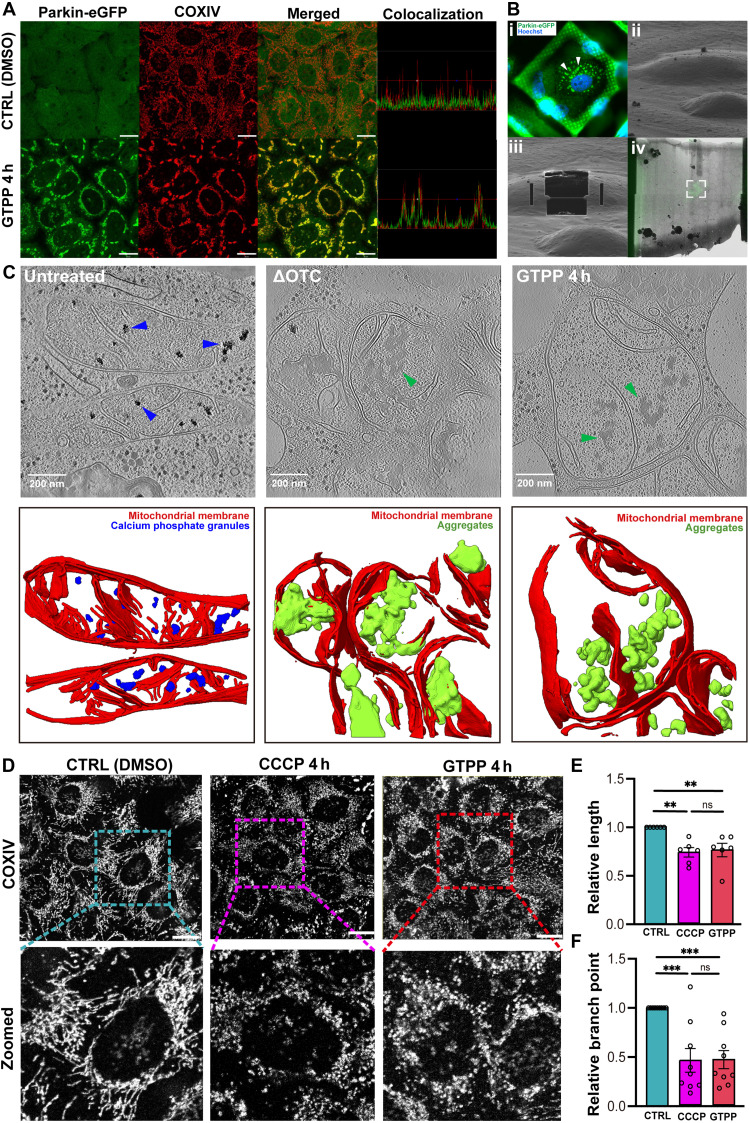
Protein folding stress induced morphological change of mitochondria. (**A**) Representative immunofluorescence images showing Parkin-eGFP (green) and the mitochondrial marker COXIV (red) in HeLa cells. Cells were treated with dimethyl sulfoxide (DMSO) for 4 hours (h) (top) or GTPP for 4 hours (bottom). Colocalization between Parkin-eGFP and COXIV was assessed using fluorescence intensity profiles. Scale bars, 20 μm. (**B**) Cryo-FIB milling workflow for GTPP-treated HeLa-Parkin-eGFP cells. (i) Cryo-FM image before milling, showing Parkin-eGFP (green) and Hoechst (blue) labeling the nucleus. (ii) Cryo-FIB/SEM image of the same cell before milling. (iii) Cryo-FIB/SEM image after lamella preparation. (iv) Correlated fluorescence image overlaid onto the SEM montage of the milled lamella, showing alignment of Parkin-eGFP signal within the targeted region (white box). (**C**) Representative tomographic slices (top) and corresponding segmentation images (bottom) of untreated, truncated OTC–expressing, and GTPP-treated cells. Blue arrowheads indicate electron-dense calcium phosphate granules (untreated), and green arrowheads indicate amorphous aggregates (OTC-expressing and GTPP condition). Scale bars, 50 nm. (**D**) Mitochondrial morphology changes upon stressor treatment. Representative immunofluorescence images of HeLa cells stained for COXIV following treatment with vehicle (CTRL; DMSO), CCCP (4 hours), or GTPP (4 hours). Zoomed-in views highlight mitochondrial network organization under each condition. (**E** and **F**) Quantification of mitochondrial morphology parameters. Relative mitochondrial length (E) and relative number of branch points across treatment conditions (F). Biological replicates = 9. Data are presented as means ± SD. One-way analysis of variance (ANOVA) followed by post hoc Tukey’s test was performed to assess statistical significance. **P < 0.01; ***P < 0.001. ns, not significant.

To visualize mitochondria under folding stress, we used a correlated cryo-fluorescence (cryo-FM) and electron microscopy (EM) approach. HeLa cells stably expressing Parkin–enhanced green fluorescent protein (eGFP) were treated with GTPP, a known inhibitor of mtHsp90, to induce the folding stress (*2*, *4*, *17*). Since GTPP-induced accumulation of unfolded protein recruits Parkin to the mitochondrial membrane (*17*, *18*), we could readily identify affected mitochondria by tracking Parkin-eGFP puncta. After a 4-hour GTPP treatment, we observed a pronounced increase in Parkin-eGFP puncta associated with stressed mitochondria, whereas no such puncta were detected in untreated controls (Fig. 1A). Supporting this observation, 84.2% of mitochondria in GTPP-treated cells were colocalized with Parkin-eGFP puncta (Fig. 1A and fig. S1A). Morphologically, the vast majority of these mitochondria exhibited fragmentation, a hallmark of mitochondrial stress (*19*), indicating that most mitochondria in our dataset were affected by protein-folding stress (Fig. 1A).

To adapt this system for cryo-ET, we used cryo–focused ion beam and scanning EM (cryo-FIB/SEM) milling on regions containing Parkin-eGFP puncta (Fig. 1B and fig. S2). Following the milling process, cryo-FM confirmed that the presence of these puncta remains on the acquired lamellae (Fig. 1B). Subsequent cryo-ET imaging detected prominent amorphous aggregates in the mitochondrial matrix, which were not observed in untreated cells. These aggregates are distinguishable from calcium phosphate granules, structures commonly observed in mitochondria, by their irregular morphology and lower electron density (Fig. 1C). To quantify these aggregates, we segmented truncated mitochondria (due to FIB milling) and associated aggregates and subsequently measured their volumes. These amorphous aggregates were observed in 87% of individual mitochondria, which occupied 4.2% of segmented mitochondrial volume. They were randomly distributed within the mitochondrial matrix and exhibited irregular morphologies with no defined structure (Fig. 1C). Some aggregates appeared continuous and elongated, while others were fragmented. They exhibited a rounded shape and smooth texture, distinguishing them from more ordered protein assemblies such as fibrils or crystalline arrays (Fig. 1C) (*20*, *21*). To validate that the amorphous structures observed under folding stress were protein aggregates formed by the accumulation of unfolded proteins (*18*, *22*), we examined mitochondria expressing a truncated form of ornithine transcarbamylase (OTC), a well-established model of misfolded proteins in the mitochondrial matrix (*18*, *22*). This analysis revealed similar amorphous aggregates within the matrix, supporting the conclusion that the aggregates observed under folding stress represent misfolded protein aggregates.

Abnormal cristae structures were also frequently observed in tomograms of mitochondria under folding stress. To quantify these alterations, we measured the cristae surface area normalized to the corresponding mitochondrial volume (fig. S1B). Compared to the untreated group, the cristae surface area was significantly reduced, indicating disruption of inner membrane architecture. Mitochondrial fragmentation was further assessed by measuring mitochondrial length and the number of branch points using fluorescence microscopy (Fig. 1D). Cells under folding stress exhibited pronounced mitochondrial fragmentation, reaching levels comparable to those observed in cells treated with carbonyl cyanide *m*-chlorophenyl hydrazone (CCCP) (Fig. 1, E and F), a well-established inducer of mitochondrial fragmentation via membrane potential uncoupling (*23*). Collectively, these features highlight the morphological and structural characteristics of the amorphous aggregates and ultrastructural change of mitochondria under folding stress (*20*, *21*).

### Folding stress remodels the molecular organization of the mtHsp60-Hsp10 complex

**Fig. 2.**
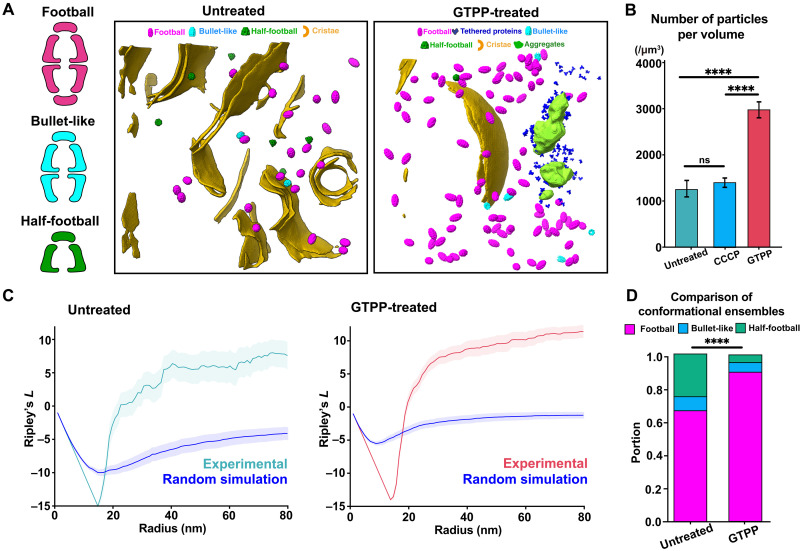
Spatial distribution and conformational dynamics of mtHsp60-Hsp10 complexes in response to folding stress. (**A**) Representative segmented tomograms from untreated and GTPP-treated cells showing mitochondrial membranes (gold), aggregates (green), and mtHsp60-Hsp10 complexes classified into football (magenta), bullet-like (cyan), and half-football (green) conformations. Tethered proteins (dark blue) and cristae (orange) are also shown in the GTPP-treated group. (**B**) Quantification of mtHsp60-Hsp10 particle concentrations in untreated (*n* = 152 tomograms), CCCP-treated (*n* = 85 tomograms), and GTPP-treated (*n* = 165 tomograms) cells. Data are presented as means ± SEM. One-way ANOVA followed by Tukey’s post hoc test was used for statistical analysis. (**C**) Ripley’s *L* function analysis of spatial clustering for mtHsp60-Hsp10 complexes in untreated (left) and GTPP-treated (right) groups, compared to random simulations. (**D**) Comparison of the relative proportions of mtHsp60-Hsp10 conformational states between untreated and GTPP-treated cells. Multivariate ANOVA was performed to assess statistical significance. ****P < 0.0001.

Given the critical role of mitochondrial chaperones in maintaining protein quality under folding stress (*2*, *4*, *17*), we performed high-magnification cryo-ET, enabling detailed spatial and structural analysis at molecular level. Using a template-based matching strategy informed by a curated set of mitochondrial chaperones and iterative particle sorting and classification (*24*), we identified a significant number of mtHsp60-Hsp10 complexes with football, half-football, and bullet-like conformation (Fig. 2A and fig. S3). We also observed smaller protein-like densities in our tomograms, some of which appeared to be tethered to the aggregates (fig. S4). However, because of their small size and low-resolution features, we could not confidently assign them to specific chaperones with known structural signatures. Accordingly, our subsequent spatial analysis focused specifically on the mtHsp60-Hsp10 complex.

Previous studies have reported elevated levels of mtHsp60-Hsp10 complexes in response to folding stress (*2*, *4*, *17*). To examine that the folding stress increases the levels of mtHsp60-Hsp10 within mitochondria, we quantified the number of particles including every conformation within measurable mitochondrial volumes in untreated, GTPP-treated, and CCCP-treated groups. CCCP induces mitochondrial stress without activating the mtUPR (*2*), allowing us to assess whether mtUPR activation is specifically required for mtHsp60-Hsp10 up-regulation. As a result, under folding stress, GTPP-treated cells exhibited an approximately twofold increase in mtHsp60-Hsp10 abundance compared to untreated and CCCP-treated cells (Fig. 2B). These results align with prior reports showing chaperone up-regulation during the mtUPR (*2*, *4*).

Previous in situ studies have reported that the tailess complex polypeptide 1-ring Complex (TRiC) forms clusters when unfolded substrates are present and that this clustering is reduced when substrate availability decreases, such as during translation inhibition (*25*). On this basis, we hypothesized that the mtHsp60-Hsp10 complex would similarly exhibit increased clustering under folding stress compared to the control group, due to the accumulation of unfolded proteins that serve as potential substrates. To test our hypothesis, we analyzed particle distribution using Ripley’s L function (*26*), where positive values indicate clustering within a defined radius and higher values reflect a greater degree of clustering. While a basal level of clustering was observed in untreated cells, the GTPP-treated group exhibited higher Ripley’s *L* values (Fig. 2C) (*27*). These results suggest that the accumulation of unfolded protein substrates under folding stress enhances the spatial clustering of the mtHsp60-Hsp10 complex.

To further investigate the relationship between aggregates and mtHsp60-Hsp10 clustering, we divided tomograms into two groups: those containing visible aggregates and those without. We then compared the concentration and spatial clustering of mtHsp60-Hsp10 particles between the two groups (fig. S5, A to D). This analysis revealed no significant differences in either particle concentration or clustering extent. Although mtHsp60-Hsp10 clustering is observed under folding stress, its distribution is not biased toward aggregate sites. These findings suggest that mtHsp60-Hsp10 clustering is primarily driven by the global accumulation of unfolded proteins, rather than by direct spatial recruitment to visible aggregates.

Previous in vitro studies have shown that the presence of substrates shifts the conformational equilibrium of mtHsp60-Hsp10 toward the football conformation (*10*), which exhibits higher folding activity than the half-football conformation, although both are functionally competent (*11*). On this basis, we reasoned that folding stress might similarly promote a shift toward the football conformation to accommodate increased chaperone demand. To assess this, we compared the conformational distribution of mtHsp60-Hsp10 between untreated and folding stress conditions (Fig. 2D). Notably, folding stress led to a significant increase in the proportion of the football conformation. These results suggest that under folding stress, the mtHsp60-Hsp10 complex increases its proportion of the football conformation as a structural response to elevated levels of unfolded proteins, thereby enhancing its folding capacity. In summary, mitochondrial proteostasis under folding stress is maintained through both an increase in chaperone levels via the mtUPR at the cellular level and through enhanced clustering and conformational switching of the mtHsp60-Hsp10 complex at the molecular level.

### In situ structural analysis of the football conformation of the mtHsp60-Hsp10 complex

**Fig. 3.**
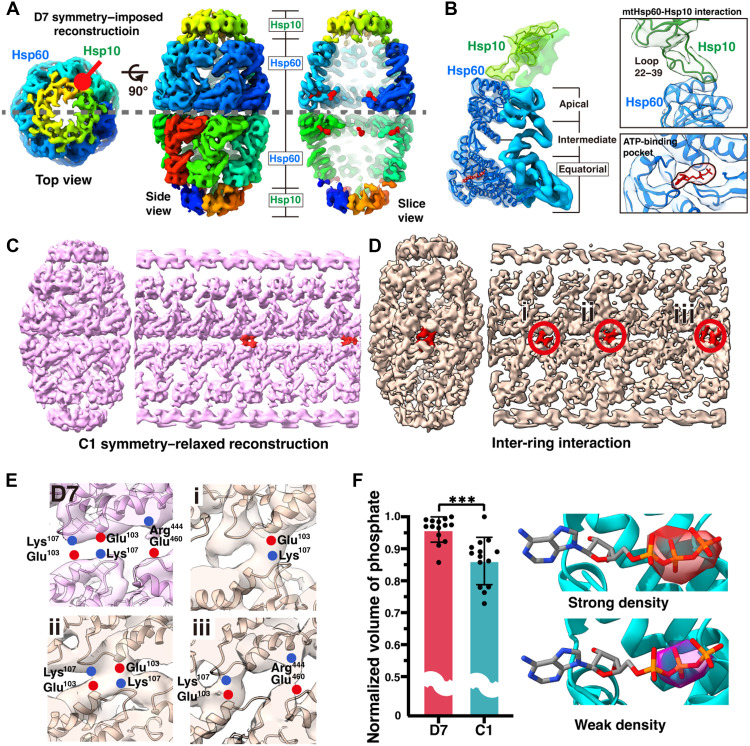
Structural details of the mtHsp60-Hsp10 complex. (**A**) Cryo-EM reconstruction of the mtHsp60-Hsp10 complex with D7 symmetry imposed (6735 particles from GTPP-treated cells). Shown are the top view (left), side view (middle), and central slice (right) of the consensus map. Hsp60 and Hsp10 subunits are colored individually to highlight symmetry, and densities corresponding to bound nucleotides are indicated in red. (**B**) Molecular model of a single mtHsp60-Hsp10 subunit derived by flexible fitting into the density map (PDB ID: 9L8P). The three structural domains of Hsp60—apical, intermediate, and equatorial—are indicated. Insets highlight (top) the interaction interface between Hsp10 and the apical domain of Hsp60 via loops 22 to 39 and (bottom) the ATP-binding pocket within the equatorial domain, with ATP shown in red. (**C**) Cryo-EM density of the mtHsp60-Hsp10 complex with C1 symmetry relaxed, showing the consensus map (left) and an unwrapped view (right). Red densities highlight asymmetric features at the inter-ring interface. (**D**) Focused classification reveals strong inter-ring densities (left), with the corresponding unwrapped view (right) showing multiple discrete sites (i to iii) of enriched inter-ring contacts (red circles). (**E**) Putative inter-ring salt bridge interactions identified at the extra density regions in the C1 map. Residue interactions are compared with the D7 map (left) and three positions (i to iii) in the C1 map. (**F**) Relative quantification of nucleotide-attributable density volumes between D7- and C1-symmetrized reconstructions. Data are presented as means ± SD (*n* = indicated particles), and statistical significance was assessed using unpaired *t* tests (***P < 0.001). Right: Examples of strong versus weak nucleotide densities visualized at identical thresholds.

To characterize the detailed in situ structural properties of the mtHsp60-Hsp10 complex under folding stress, we performed subtomogram averaging using particles extracted from stressed mitochondria. We focused primarily on the football conformation, as the half-football and bullet-like conformations were present at low abundance, yielding poorly resolved reconstructions. The football conformation analysis resulted in a map at 7.4-Å resolution under D7 symmetry (Fig. 3A and fig. S3). The structure exhibits the classical features of a football shaped group I double-ring chaperonin: 14 mtHsp60 subunits arranged in two stacked rings of seven each, capped by a heptameric mtHsp10 (*10*, *11*, *28*). The in situ structures closely resemble a previously reported in vitro cryo-EM structure of a purified mtHsp60 complex in a similar football-shaped arrangement (*10*, *28*). Flexible fitting of the in vitro model [Protein Data Bank (PDB) ID: 8G7N) into our in situ map revealed strong agreement, with an ~0.5-Å root mean square deviation value confirming consistency at both the overall architecture and secondary structure levels (Fig. 3A). This structural agreement, obtained within intact mitochondria under stress, provides direct evidence that key mechanistic features of the mtHsp60-Hsp10 system (*10*) are preserved and actively used in situ to manage unfolded protein load (Fig. 3B).

To further explore asymmetric features of the in situ mtHsp60-Hsp10 complex, we reconstructed symmetry-relaxed structures at an 8.1-Å resolution (Fig. 3C). While the overall structural features remained consistent with the symmetry-imposed map, relaxing symmetry revealed notable asymmetry in the inter-ring connectivity, suggesting dynamic variability in subunit interactions. We then performed focused classification targeting the equatorial region where the two rings interact (fig. S3). This analysis uncovered a distinct structure with additional density at the inter-ring interface (Fig. 3, D and E). With the fitted model, we identified the extra density as potential salt bridges between residues Lys^103^ and Glu^107^, as well as Arg^444^ and Glu^460^ (Fig. 3E). These features, which were less apparent in the symmetry-imposed map, became more discernible when comparing subtracted maps between the C1 and D7 structures, highlighting the asymmetric distribution of inter-ring interaction (Fig. 3E). The critical role of Lys^107^ in stabilizing the football conformation and regulating inter-ring allostery of GroEL/ES (*11*, *29*), together with the increased football population under folding stress, suggests a potential role for inter-ring communication in promoting or stabilizing the functionally active conformation of mtHsp60-Hsp10.

We also examined heterogeneity in nucleotide occupancy within the adenosine 5′-triphosphate (ATP)–binding pockets using both C1 and D7 maps. Since cryo-EM maps represent potential maps derived from signal averaging (*30*, *31*), we compared relative volumes to estimate nucleotide occupancy in the in situ mtHsp60-Hsp10 complex. As previously reported, phosphate-attributable density is more pronounced in cryo-EM maps, enabling quantitative comparison of relative volume intensities across Hsp60 subunits (*30*). In the D7 symmetry-imposed map, ATP-binding pocket volumes were consistent across all 14 subunits. However, significant variability was observed in the C1 symmetry–relaxed map, indicating uneven nucleotide binding across subunits (Fig. 3F). We normalized volume densities by the maximum volume among 14 subunits, enabling direct comparison across subunits (Fig. 3F). Our analysis suggests that, in situ, the ATP-binding sites in the Hsp60-Hsp10 complex are not fully occupied, with ~12 of the 14 nucleotide-binding pockets showing evidence of occupancy, which indicates that fully occupied nucleotide is not necessary for its function and formation of football complex (Fig. 3F) (*31*). However, because of the resolution limitations of the C1 map, this observation should be interpreted with caution and may require further structural validation.

Collectively, our in situ structural analysis highlights the biological relevance of the mtHsp60-Hsp10 complex, confirming that its essential architecture—featuring asymmetric intersubunit interactions and dynamic nucleotide binding—is preserved under physiological conditions. These features are critical for the chaperonin’s functional adaptability and efficiency in its native environment (*10*).

### Molecular contacts of encapsulated substrate inside the mtHsp60-Hsp10 chamber

**Fig. 4.**
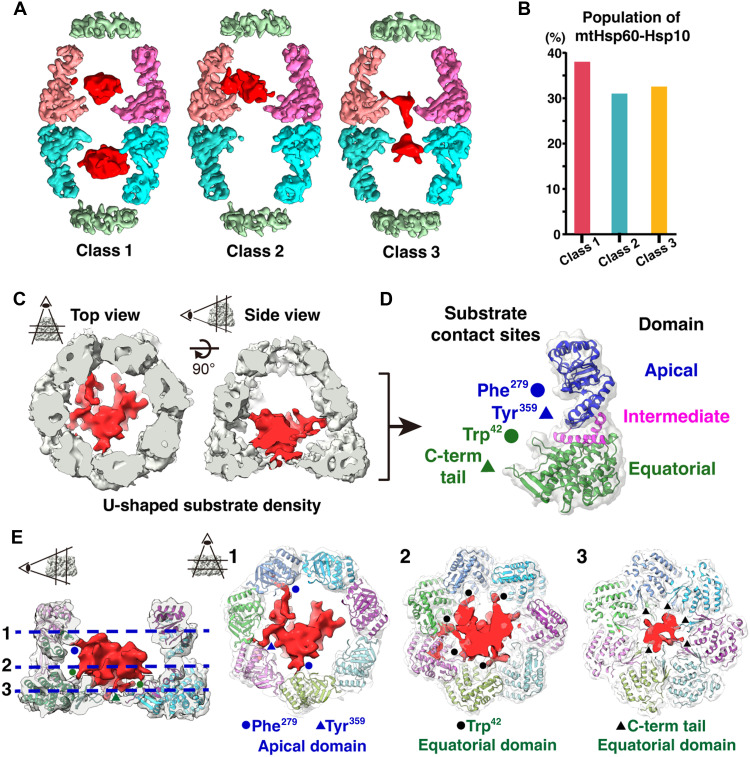
Substrate engagement within the mtHsp60-Hsp10 complex. (**A**) Cryo-EM maps of three representative classes of the mtHsp60-Hsp10 complex containing distinct substrate densities. Class 1 features asymmetric substrate occupancy—bulky density in one chamber and sparse density in the other. Class 2 contains bulky densities in both chambers, while class 3 contains sparse densities in both chambers. A Gaussian filter was applied to substrate densities (red) for visualization. (**B**) Relative population distribution of the three mtHsp60-Hsp10 conformational classes. (**C**) Top and side views of the U-shaped substrate density (red) inside the mtHsp60-Hsp10 complex (gray). (**D**) Mapping of four substrate contact sites on the mtHsp60 model. The apical domain is shown in blue, intermediate in magenta, and equatorial in green. Contact residues include Phe^279^ (apical), Tyr^359^ (apical), Trp^42^ (equatorial), and the C-terminal tails (equatorial). (**E**) Side view (left) and corresponding slice views (right) of three vertical layers of substrate contact.

To further explore how mtHsp60-Hsp10 interacts with its substrate in situ, we conducted three-dimensional (3D) classification focusing on both folding chambers (fig. S3), using a methodology similar to that used in in situ studies of GroEL (*32*). This analysis revealed various density shapes within the chambers, allowing us to categorize mtHsp60-Hsp10 into three distinct classes based on the presence and shape of densities: Class 1 featured one chamber occupied by a bulky density and the other by a sparse density, class 2 had both chambers occupied by bulky densities, and class 3 exhibited both chambers occupied by sparse densities (Fig. 4A). These observations are consistent with previous studies indicating that group I chaperonins can use both folding chambers to simultaneously encapsulate substrates. In our study, the populations and spatial locations of complexes containing one bulky density versus two bulky densities did not exhibit significant differences in the tomograms (Fig. 4B and fig. S6).

Subsequently, we investigated the molecular contacts between mtHsp60-Hsp10 and its encapsulated substrate to understand how mtHsp60 stabilizes the substrate in situ. By performing 3D classification focused on a single folding chamber, we obtained a map with localized density on the side of the chamber, revealing significant contact sites with mtHsp60-Hsp10 (Fig. 4C). Our structural analysis highlights key aspects of the interaction between the substrate and the mtHsp60 ring, while no direct contact with Hsp10 was observed. Using flexible fitted model (PDB ID: 9L8P), we identified that the observed substrate density is directly associated with specific mtHsp60 subunits at several sites involving the hydrophobic residues Phe^279^, Tyr^359^, and Trp^42^ in the S loop (Fig. 4, D and E, and fig. S7), which are highly conserved in group I chaperonins (*15*). Phe^279^ and Tyr^359^ are situated on helices within the apical domain of mtHsp60 and interact with the upper region of the substrate, whereas Trp^42^ is located near the equatorial domain, appropriately positioned to stabilize the lower part of the substrate (Fig. 4E). These aromatic residues extend toward the center and align along prominent hydrophobicity belts at the middle and lower levels of the mtHsp60 ring, thereby promoting effective interactions with the substrate. In addition, we observed extra density at the base of the chamber, primarily located in the region of the unstructured C-terminal tails (residues 523 to 547) of mtHsp60 subunits, which displays connectivity to the substrate density. These hydrophobic interactions and C-terminal tail interactions have been previously described in in vitro studies of group I chaperonins GroEL:ES (*15*) and mtHsp60-Hsp10 (*10*). Our findings extend these insights to the mtHsp60-Hsp10 system, highlighting that the structural and functional characteristics of mtHsp60-Hsp10 are preserved under physiological conditions.

### Coordinated mitochondrial quality control by chaperones and mitophagy

**Fig. 5.**
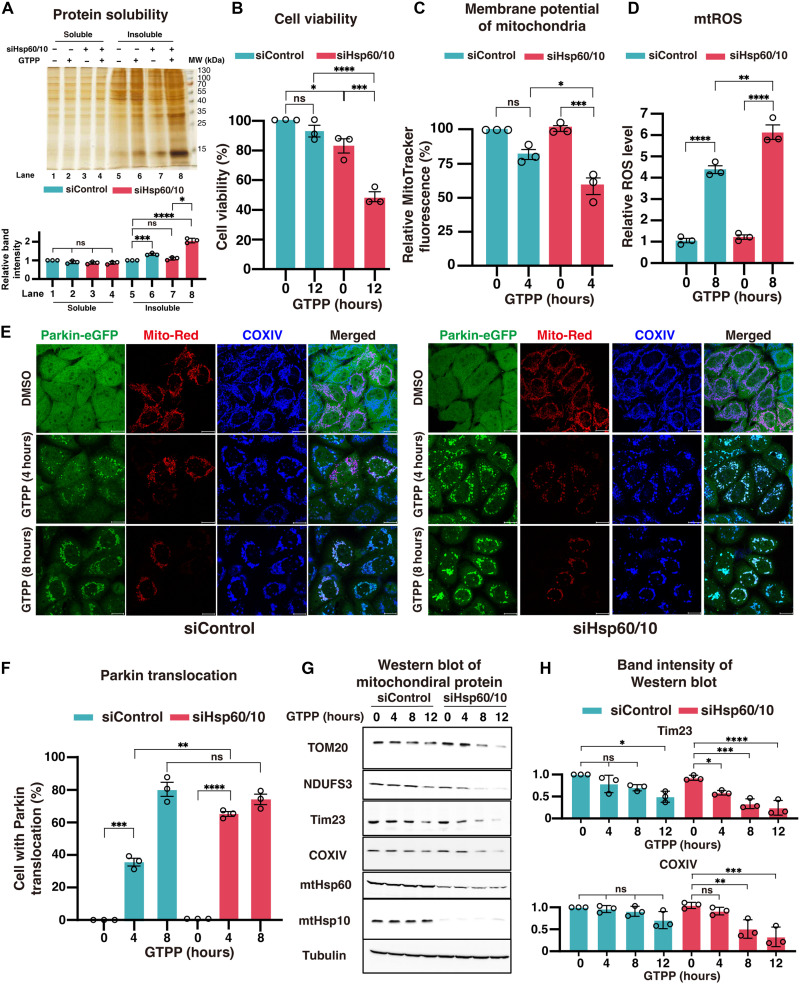
Phenotypic consequences of mtHsp60-Hsp10 complex knockdown under folding stress. (**A**) Silver staining analysis of soluble and insoluble protein fractions from siControl and siHsp60/10-treated cells, with or without GTPP treatment. MW, molecular weight. (**B** to **D**) Quantification of cellular stress markers following GTPP-induced folding stress under control and knockdown conditions: Cell viability (B), mitochondrial membrane potential (MitoTracker fluorescence) (C), and mtROS levels (D). Data are shown as means ± SD. Statistical significance was assessed using unpaired *t* tests or one-way ANOVA with Tukey’s post hoc test. *P < 0.05; **P < 0.01; ***P < 0.001; ****P < 0.0001. (**E**) Representative immunofluorescence images of HeLa cells under increasing folding stress, comparing siControl (left) and siHsp60/10 knockdown (right) conditions. Cells were treated with DMSO or GTPP for 4 or 8 hours and stained for Parkin-eGFP (green), MitoTracker Red (red), and COXIV (blue). Merged images highlight the mitochondrial network and Parkin puncta localization. Scale bars, 20 μm. (**F**) Quantification of Parkin translocation in siControl and siHsp60/10 cells treated with GTPP for 0, 4, or 8 hours. Parkin puncta–positive cells were counted as a percentage of total cells. (**G**) Western blot analysis of mitochondrial proteins (TOM20, NDUFS3, Tim23, COXIV, mtHsp60, and mtHsp10) following GTPP treatment for the indicated times. Tubulin was used as a loading control. (**H**) The intensity profiles of the Western blot bands for Tim23 and COXIV were presented. All of the intensities were normalized by tubulin. All experiments were performed with three biological replicates. Data are presented as means ± SD. One-way ANOVA followed by Tukey’s post hoc test for multiple comparisons was performed to assess statistical significance. *P < 0.05; **P < 0.01; ***P < 0.001; ****P < 0.0001.

Our in situ spatial and structural analysis revealed that mtHsp60-Hsp10 complexes are locally concentrated around the aggregates and encapsulate unfolded proteins. To confirm whether these mtHsp60-Hsp10 complexes are involved in mitigating folding stress, we measured protein solubility following knockdown of mtHsp60-Hsp10 complex under folding stress. We found that insoluble fractions were significantly elevated in knockdown cells under folding stress (Fig. 5A). We then assessed the stress associated with the accumulation of unfolded proteins by measuring cell viability, MitoTracker fluorescence intensity, which indicates membrane potential and mtROS. Under untreated conditions, viability was decreased 17% in knockdown cells, but there were no significant differences in MitoTracker intensity or mtROS between knockdown and control cells (Fig. 5, B to D). In terms of viability and MitoTracker intensity, GTPP treatment did not induce any significant changes in control cells (Fig. 5, B and C). However, in knockdown cells, GTPP treatment resulted in a 47% decrease in cell viability and 27% reduction in MitoTracker intensity compared to control cells under folding stress (Fig. 5, B and C). We further confirmed that these resulting phenotypes were not attributable to knockdown-induced apoptosis by assessing apoptotic marker levels (fig. S8, A and B). Similarly, mtROS levels were 40% higher in knockdown cells compared to control cells under folding stress (Fig. 5D). To examine the influence of mtHsp60-Hsp10 knockdown to morphological changes, we quantified the volumes of aggregates and surface area of cristae. As a result, knockdown of mtHsp60-Hsp10 induced formation of larger aggregates (fig. S9, A and B), but there were no significant differences in cristae remodeling (fig. S9B). These findings demonstrate that, in the insufficiency of mtHsp60-Hsp10, cells and mitochondria become highly vulnerable to folding stress and emphasize the importance of mtHsp60-Hsp10 complex to mitigate folding stress.

Previous studies have shown that protein-folding stress triggers PTEN-induced kinase 1 (PINK1)/Parkin-mediated mitophagy, which selectively degrades damaged mitochondria (*17*, *18*). To confirm that mitophagy-related phenotypes were present in our system, we validated colocalization between LC3 and Parkin-eGFP (fig. S9C) and observed lysosomes containing mitochondrial content using targeted cryo-ET at regions colocalized with mitochondria-targeted Keima (mt-Keima) and LysoTracker (fig. S9, D and E). Considering increased cellular stress upon chaperone knockdown and PINK1/Parkin-mediated mitophagy under folding stress, we hypothesized that mitochondria initially respond to folding stress by up-regulating chaperones, including the mtHsp60-Hsp10 complex, and when the stress level exceeds the capacity of the chaperone system, these overburdened mitochondria are selectively degraded via PINK1/Parkin-mediated mitophagy.

To test this hypothesis, we knocked down mtHsp60 and mtHsp10 and examined Parkin translocation, an early event in PINK1/Parkin-mediated mitophagy. Under GTPP-untreated conditions, neither the knockdown nor control groups exhibited any Parkin translocation (Fig. 5E). After 4 hours of GTPP treatment, however, Parkin translocation was 75% higher in knockdown cells compared to control cells, and by 8 hours, translocation reached saturation at ~80% in both knockdown and control groups (Fig. 5, E and F). To confirm that this increase in Parkin translocation led to mitophagy, we next measured mitochondrial degradation by monitoring the quantity of mitochondrial proteins and mt-Keima analysis. Consistent with the Parkin translocation data, knockdown cells exhibited rapid and significant mitochondrial degradation via PINK1/Parkin-mediated mitophagy in both experiments (Fig. 5, G and H). These results support our hypothesis that mtHsp60-Hsp10 chaperones help mitigate mitochondrial folding stress and that once this system is overwhelmed, the damaged mitochondria are selectively eliminated through PINK1/Parkin-mediated mitophagy.

## DISCUSSION

Using correlated cryo-ET, we directly visualized mitochondrial responses to folding stress, revealing characteristic morphological changes, including mitochondrial fragmentation and cristae disorganization (Fig. 1, C to E). Under these conditions, mtHsp60-Hsp10 complexes formed discrete clusters and remodeled their conformational ensembles within the mitochondrial matrix, suggesting stress-induced self-organization of chaperone networks (Fig. 2, A to D). Consistently, knockdown of mtHsp60-Hsp10 followed by folding stress significantly increased aggregates and cellular stress compared to control cells. These observations, together with previous studies (*2*, *4*), led us to propose a mechanism by which the chaperone network responds to mitochondrial folding stress (fig. S10). When proteostasis is disrupted, unfolded proteins accumulate, forming amorphous aggregates and increasing mtROS levels, which collectively impair mitochondrial protein import (*4*). This condition activates transcriptional stress responses that up-regulate chaperone networks including mtHsp60 and mtHsp10 (Fig. 2B) (*2*, *4*). The increased abundance of these chaperones, along with stress-induced clustering and cooperative conformational changes, enhances their ability to refold unfolded proteins (*11*). When folding stress exceeds the buffering capacity of this system, persistent dysfunction triggers PINK1/Parkin-mediated mitophagy, thereby removing irreversibly damaged mitochondria and preserving cellular homeostasis (*17*, *18*).

Our in situ structural analysis further revealed previously uncharacterized conformational features of the mtHsp60-Hsp10 complex under stress. Subtomogram averaging showed that the complex retains its canonical double-ring architecture and exhibits dynamic conformational heterogeneity similar to that observed in vitro, including football and half-football states (*10*, *11*). Notably, we also identified a minor population resembling a bullet-like conformation, consistent with transient intermediates proposed in earlier studies (*33*). These observations suggest that conformational cycling of the chaperonin complex occurs in situ and may be modulated by stress-induced changes in inter-ring interactions (Fig. 3, C to E). Under folding stress, we also observed structural asymmetry and substrate-dependent contacts involving conserved hydrophobic residues—such as Phe^279^, Tyr^359^, and Trp^42^—lining the inner chamber, indicating active substrate engagement (Fig. 4, D and E). These findings are consistent with models in which substrate binding stabilizes specific chaperonin states and alters subunit communication, as shown in GroEL systems (*34*, *35*). Together, these structural insights point to a dynamic, cooperative mechanism by which the mtHsp60-Hsp10 complex adapts to buffer proteotoxic stress.

While these findings offer important clues into the in situ operation of mitochondrial chaperones, our study also highlights several limitations. Because of the limited signal-to-noise ratio and intrinsic challenges of cryo-ET, we were unable to resolve high-resolution asymmetric structural details of inter-ring cooperativity or to discriminate among substrate-engaged intermediates. Furthermore, although previous studies suggest that general chaperones such as mtHsp70 are up-regulated alongside mtHsp60-Hsp10 and contribute to mitochondrial homeostasis (*2*, *4*, *22*), their cooperative roles remain poorly understood in structural terms. In our dataset, we detected small protein–like densities decorating the aggregates (Fig. 2B), which may represent additional chaperone components but could not be definitively identified. This raises the possibility that mtHsp60-Hsp10 acts within a broader stress-induced chaperone network that is dynamically reorganized in space and time (*36*). To accurately interpret structure-function relationships in situ, future studies should incorporate time-resolved analyses of individual mitochondria, enabling discrimination between those that successfully mitigate folding stress and those committed to mitophagy. Combining this approach with high-resolution structural methods and molecular labeling will be critical to elucidate how distinct mitochondrial fates are coupled to chaperone network remodeling.

## MATERIALS AND METHODS

### Cell culture

HeLa cells expressing Parkin-eGFP were cultured in Dulbecco’s modified Eagle’s medium (Gibco) supplemented with 10% fetal bovine serum (FBS; Gibco), 1% penicillin/streptomycin (Gibco), and puromycin (1 μg/ml; Sigma-Aldrich) at 37°C in a humidified atmosphere composed of 5% CO_2_. To ensure consistency of results, the number of passages was kept below 15, and cell confluence was maintained at less than 80%.

### Colocalization analysis of mitochondria and Parkin in mammalian cells

Confocal images were acquired using a Zeiss LSM confocal microscope equipped with appropriate laser lines and a 40× water-immersion objective. Image processing and analysis were conducted using ZEN software (Zeiss). Colocalization between mitochondria and Parkin was assessed by calculating the Pearson’s correlation coefficient and Manders’ overlap coefficient using the colocalization analysis module in ZEN. Uniform background subtraction and thresholding were applied across all samples to minimize nonspecific signals. For quantification, at least 10 randomly selected cells per condition were analyzed. Colocalization data are expressed as means ± SEM. Statistical analyses were performed using GraphPad Prism software, and significance was determined using appropriate tests with a threshold of *P* < 0.05.

### Mitochondrial morphology analysis in mammalian cells

Mitochondrial morphology was quantitatively analyzed from confocal fluorescence images using a custom Python-based pipeline (v3.10). Grayscale images were binarized using a fixed intensity threshold (value = 200) to segment individual mitochondrial structures. The binary images were subsequently skeletonized to extract 1-pixel-wide medial axes representing the mitochondrial network. Discrete mitochondria were identified as connected components within the skeletonized image. Mitochondrial length was calculated by counting the number of pixels per skeleton and converting the value to micrometers using a calibrated pixel size of 0.238 μm (based on a 20-μm scale bar corresponding to 84 pixels). Branch points were defined as skeleton pixels with more than two neighbors in an eight-connected neighborhood and were quantified per image field. To ensure consistent sampling across conditions, the number of mitochondria analyzed per image was normalized by random subsampling. For spatial analysis of mitochondrial architecture, each image was divided into nine equal horizontal bins, and morphological metrics—including mean length, standard deviation, and branch point count—were calculated within each bin.

### Small interfering RNA sequences information

We used the following small interfering RNA (siRNA) sequences to target human Hsp60 and Hsp10: Hsp60 siRNA, GUGUUGAAGGAUCUUUGAU=tt; Hsp10 siRNA, GAAAUCUUUCGUCAUGUAA=tt.

### On-grid culture and cryo-specimen preparation

Quantifoil 1/4 200 mesh Au grids (Quantifoil) were glow discharged for 30 s and sterilized with ultraviolet light for 1 hour. The grids were then coated with fibronectin (50 μg/ml) for 1 hour, washed three times with phosphate-buffered saline (PBS), and placed onto 35-mm imaging dishes (Mattek). Approximately 150,000 cells were seeded onto the grids and allowed to adhere overnight. This resulted in an optimal confluency of approximately 300 cells per grid. Subsequently, cells were treated with 10 μM GTPP for 4 hours.

For knockdown samples, cells cultured on 35-mm imaging dishes (Mattek) were transfected with siRNA using Lipofectamine RNAiMAX (Invitrogen) following the manufacturer’s protocol. At 24 hours posttransfection, cells were detached and seeded onto precoated EM grids as described above. At 48 hours posttransfection, cells were treated with 10 μM GTPP for 4 hours.

For truncated OTC expressed samples, cells cultured on 35-mm imaging dishes (Mattek) were transfected with pCAGGS-truncated OTC, which was a gift from N. Hoogenraad (plasmid #71877, Addgene), using Lipofectamine 3000 (Invitrogen) following the manufacturer’s protocol. At 24 hours posttransfection, cells were detached and seeded onto precoated EM grids as described above. At 48 hours posttransfection, cells were treated with 10 μM GTPP for 4 hours.

For mt-Keima expressed samples, cells cultured on 35-mm imaging dishes (Mattek) were transfected with pcDNA–mt-Keima, which was a gift from J. Yun (Dong-A University, Korea), using Lipofectamine 3000 (Invitrogen) following the manufacturer’s protocol. At 24 hours posttransfection, cells were detached and seeded onto precoated EM grids as described above. At 48 hours posttransfection, cells were treated with 10 μM GTPP for 4 hours. LysoTracker Deep Red (Thermo Fisher Scientific) was applied following the manufacturer’s protocol before vitrification.

Hoechst 33342 (Miltenyi Biotech) was applied following the manufacturer’s protocol before vitrification in all experiments. Five microliters of 1 μm of Dynabeads (Thermo Fisher Scientific), which were diluted in 1:100 in PBS with 10% glycerol, was added to the grids right before blotting. Grids were single-side blotted for 5 s and vitrified using an EMGP2 (Leica Microsystems). Untreated cells were prepared and vitrified under the same conditions.

### Cryo-FIB/SEM milling and cryo-ET

Cryo-FIB/SEM milling was performed using an Aquilos 2 Cryo-FIB (Thermo Fisher Scientific). For GTPP-treated cells, 3D correlation targeting Parkin-eGFP puncta was achieved using the integrated fluorescence microscopy system in Aquilos 2 and Maps 3.20 software (Thermo Fisher Scientific). To minimize charging and curtaining effects, Pt sputter coating (10 s at 10 mA) and a gas injection system (8 s per grid) were applied before milling. Lamellae were milled to a final thickness of 120 nm with decreasing FIB currents (0.5, 0.3, and 0.1 nA and 50 pA) using AutoTEM 2.4 software (Thermo Fisher Scientific). Untreated cells were milled identically, irrespective of Parkin-eGFP puncta. Lamellae were imaged using Tomography 5 software (Thermo Fisher Scientific) with a Titan Krios G4 equipped with Falcon 4i detector (Thermo Fisher Scientific) and Selectris X energy filter (Thermo Fisher Scientific). For morphological analysis, tilt series were acquired at 3.06 Å per pixel with a total exposure of 120 to 150 e^−^/Å^2^ at a defocus of 5 μm using a dose-symmetric scheme. The pretilt angle was set at ±10°, and tilt ranges spanned ±50° with 2.5° increments. For structural and spatial analysis, 1.23 Å per pixel with a total exposure of 120 to 150 e^−^/Å^2^ at a defocus range of 3.5 to 4.5 μm using a dose-symmetric scheme. The pretilt angle was set at ±10°, and tilt ranges spanned ±40° with 2° increments.

### Data processing

Preprocessing including motion correction and contrast transfer function (CTF) estimation of collected micrographs was performed by Warp 1.1.0 beta (*37*). Poor-quality micrographs were manually excluded, and tilt series stacks were generated. Fiducial-less alignment of tilt series was performed using AreTomo (version 1.3.4) (*38*). CTF estimations of aligned tilt series were calculated in Warp, and tomograms at 24.48 Å per voxel were reconstructed (*37*, *38*). A total of 15 tomograms from the control group, 48 tomograms from the GTPP-treated group, and 18 tomograms from knockdown followed by GTPP treatments were used for further analysis. For high-magnification datasets, all preprocessing steps were performed as described above, and tomograms were reconstructed at a pixel size of 9.84 Å per voxel. A total of 152 tomograms from the control group, 165 tomograms from the GTPP-treated group, and 85 tomograms from the CCCP-treated group were used for further analysis. Missing wedge-corrected tomograms were generated using IsoNet (*39*).

For the subtomogram averaging of tethered proteins, proteins were manually picked in napari (*40*). The coordinates were converted using crYOLO for compatibility with Warp (*40*, *41*). Subtomograms were extracted at 9.84 Å per voxel and filtered by 3D classification with alignments in RELION-4.0 (*42*). Filtered subtomograms were used for averaging. Molceular dynamics flexible fitting (MDFF) using the N-terminal domain of AlphaFold 3 predicted mtHsp70 (*43*) was performed using Namdinator (*44*).

For the template matching of the mtHsp60-Hsp10 complex, the previous in vitro model (PDB ID: 8G7N) was used (*10*). The PDB file was converted to an EM map using pdb2mrc (EMAN2) (*45*). Using acquired maps and tomograms, template matching was performed by pyTOM (*24*). Acquired coordinates of particles were imported onto Warp, and subtomograms at 9.84 Å per voxel were extracted. Using the extracted subtomograms, 3D classification with alignment enabled the selective identification of mtHsp60-Hsp10 football densities and effectively filtered out false positives in RELION-4.0 (*42*). To analyze mtHsp60-Hsp10 football conformation influenced by folding stress, we used particles solely from GTPP-treated tomograms. Subtomograms were reextracted at 4.92, 2.46, and 1.70 Å per voxel sequentially (*37*). Using subtomograms at 1.70 Å per voxel, consensus maps were acquired (*42*). 3D classification without alignments using the mask focused on the equatorial domain and inner chamber was performed independently, and final maps were subsequently acquired through 3D refinement (*42*). A bullet-like conformation was resolved through 3D classification without alignment, using a mask focused on the mtHsp10 region. Unwrapped maps were acquired using e2unwrap3d.py (*45*). Classes from each classification were visualized by UCSF ChimeraX (*46*). Untreated samples underwent identical processing.

For template matching of the half-football conformation, EMD-9196 was used as the reference template. Template matching was performed using the same procedure as described above. Because of the low abundance of particles adopting the half-football conformation, 3D classification with alignment was conducted on merged particles from both untreated and GTPP-treated samples. Through iterative 3D classification with alignments, genuine half-football particles were isolated and subsequently refined using C7 symmetry.

### Classification of mtHsp60-Hsp10 complex

All of the classifications were performed using particles at 1.70 Å per voxel and 3D classification without alignments. In the case of equatorial domains, we used a mask around the whole equatorial domain of both rings. We identified a distinctive class that has strong additional interaction. At last, classification using a mask around the inner chamber of the complexes yielded three different states of complexes with different volumes of substrate density. Subsequent 3D classification using a mask including one side of the chamber region revealed U-shaped substrate density within mtHsp60-Hsp10. All of the states were visualized by UCSF ChimeraX (*46*).

### Model building

The in vitro model of the mtHsp60-Hsp10 football complex (PDB ID: 8G7N) was used as the initial template for model building. Since this model incorporates a single Val^72^→Ile mutation, it was reverted to the wild-type sequence using Coot (*47*). Subsequently, the modified models were flexibly fitted into the D7 consensus map using the ISOLDE software (*48*). The fitted model was further manually adjusted in Coot (*47*) and refined in Phenix (*47*, *49*) to achieve accurate structure.

### Analysis of the nucleotide attributable density

For the analysis of phosphate density, we followed previously described procedure (*30*), and in our analysis, we measured the volume of the phosphate regions, not the whole nucleotide due to limitations in resolution. First, we set all of the total volume of the complex at 529.9 × 10^3^ Å^3^. Then, we assigned unique colors to each triphosphate in the flexible fitted model (PDB ID: 9L8P) and fit the model into the maps. Last, we segmented each phosphate density from different classes and measured their volumes. These volumes were then normalized by the maximum phosphate volume of each complex or single ring for consistency. To evaluate statistical difference, we conducted an unpaired *t* test to compare the phosphate volumes between the cis and trans rings or between the complexes.

### Segmentation of mitochondrial components, along with mapping of mtHsp60-Hsp10

Mitochondrial membrane was segmented by MemBrain (*50*), and the amorphous aggregates were manually segmented by Amira (v2023.1, Thermo Fisher Scientific). Small proteins were segmented using intensity-based segmentation in Amira. Tethered proteins were manually picked and mapped, and mtHsp60-Hsp10 complexes were mapped using subtomo2chimera (*51*). Mapped proteins and aggregates were visualized by UCSF ChimeraX (*46*).

### Measuring volume of mitochondria, aggregates, and surface area of cristae

For each tomogram, a mask encompassing the entire voxel volume within its *z* thickness was generated in RELION-4.0 (*40*). The mask was combined with the segmented mitochondrial membranes in Amira (Thermo Fisher Scientific) to define mitochondrial region of interest and to manually remove nonmitochondrial densities. The mitochondrial volume within each tomogram was then measured using Amira to ensure precise volumetric analysis. For the aggregates, using the segmented density of the aggregates, the volumes of aggregates from each experimental group were calculated using the Volume Fraction tool in Amira (Thermo Fisher Scientific). To quantify the surface area of cristae, we first segmented entire mitochondrial membranes in the tomograms. The outer double membrane was then removed using the volume edit tool in Amira, leaving only the internal cristae structures. The surface area of remaining internal cristae was then measured using the volume fraction tool in Amira.

### Statistical analysis of cristae, aggregates, and concentration of mtHsp60-Hsp10 complex

To statistically analyze ultrastructure of mitochondria, the surface area of cristae and the volume of aggregates were normalized to the corresponding mitochondrial volumes. To enhance statistical interpretation, the 1.5× interquartile range (IQR) rule was applied. For concentration analysis, particle numbers from the football, bullet-like, and half-football conformations were combined, and the total was divided by the identified mitochondrial volume in each tomogram. Concentration values were calculated individually for each tomogram and used for subsequent statistical analysis. To minimize processing errors caused from subtomogram averaging, both the 1.5× IQR rule and concentration-based weighting were applied for statistical analysis. Data were presented by GraphPad Prism software and custom Python codes.

### Calculation of Ripley’s *L* function

Ripley’s *L* function was calculated using modified Python codes of “pyORG” (*27*, *52*). A mitochondrial mask from each tomogram was applied to spatially restrict the analysis of the mtHsp60-Hsp10 complex. Combined particles from the football, bullet-like, and half-football conformations were used. To enhance the validity of the analysis, the standard error of the mean (SEM) was calculated using concentration-based weighting.

### Immunoblotting analysis

HeLa cells stably expressing GFP-Parkin were seeded in six-well plates and transfected with siRNA using Lipofectamine RNAiMAX (Invitrogen) according to the manufacturer’s instructions. After 48 hours, cells treated with 10 μM GTPP (HY-102007A, MedChemExpress) for 4, 8, and 12 hours. Immunoblot analysis was performed on mitochondrial proteins using the following antibodies: anti-Tom20 (4206, Cell Signaling Technology; dilution, 1:1000), anti-NADH:ubiquinone oxidoreductase core subunit s (NDUFS3) (ab14711, Abcam; dilution, 1:1000), anti-translocase of the inner mitochondrial membrane 23 (Tim23) (ab230253, Abcam; dilution, 1:1000), and anti-cytochrome c oxidase subunit IV (4850, Cell Signaling Technology; dilution, 1:1000). Anti-tubulin was used as a loading control, and anti-mtHsp60 (ab46798, Abcam; dilution, 1:1000) was used to assess the efficiency of mtHsp60 knockdown. Cells were lysed with radioimmunoprecipitation assay buffer [50 mM tris (pH 8.0), 150 mM NaCl, 0.5% sodium deoxycholate, 1% NP-40, 0.1% SDS, 50 mM NaF, 1 mM sodium vanadate, 1 mM phenylmethylsulfonyl fluoride, leupeptin (10 μg/ml), and pepstatin A (1 μg/ml)]. Total protein concentration was determined using the BCA Protein Assay Kit (Pierce). Protein lysates were separated by SDS–polyacrylamide gel electrophoresis and transferred to a 0.2-μm nitrocellulose membrane (GE Healthcare) for immunoblotting according to standard procedures. We used secondary horseradish peroxidase (HRP) antibodies: HRP-rabbit (111-035-144, Jackson ImmunoResearch; dilution, 1:5000) and HRP-mouse (115-035-146, Jackson ImmunoResearch; dilution, 1:5000). The blots were visualized using a LAS-4000 imager (Fujifilm) and Multi Gauge v.3.0 (Fujifilm), and band intensity was quantified using ImageJ software.

### Immunofluorescence analysis of Parkin translocation to mitochondria

For immunofluorescence analysis, HeLa cells stably expressing GFP-Parkin were seeded on 18-mm coverslips in 12-well tissue culture plates. After 48 hours of siRNA and Mito-dsRed (#632421, Invitrogen) transfection using Lipofectamine 2000 (Invitrogen) according to the manufacturer’s instructions, cells were fixed with 4% paraformaldehyde (BIOSESANG) for 15 min, permeabilized with ice-cold 100% methanol for 20 min, and blocked with a solution containing 4% bovine serum albumin and 1% normal goat serum in 0.1% Triton X-100 in PBS for 1 hour at room temperature. Primary antibodies (anti-COXIV, 4850, Cell Signaling Technology; dilution, 1:100) were diluted in the blocking solution and incubated with the cells overnight at 4°C. Following four washes with PBST (0.1% Triton X-100 in PBS), cells were incubated with secondary antibodies (Alexa Fluor 647, A21245, Thermo Fisher Scientific; dilution, 1:100) for 1 hour at room temperature. The antibody-labeled cells were then washed six times with PBST and mounted on slides using a mounting solution [1,4-diazabicyclo[2.2.2]octane (100 mg/ml) in 90% glycerol]. Immunofluorescent images were captured using an LSM710 laser scanning confocal microscope (Carl Zeiss).

### Measurement of ROS levels, cell viability, and mitochondrial potential

HeLa cells stably expressing GFP-Parkin were seeded in six-well plates and transfected with siRNA using Lipofectamine RNAiMAX (Invitrogen) according to the manufacturer’s protocol. After 48 hours, cells were stained to measure ROS levels, cell viability, and mitochondrial membrane potential. For ROS measurement, cells were incubated with 1 μM MitoSOX Red (Invitrogen) in Hanks’ balanced salt solution (HBSS; WELGENE) for 30 min at 37°C in a humidified atmosphere with 5% CO_2_. For cell viability, cells were stained with 5 μM ethidium homodimer-1 (EthD-1; Invitrogen) in PBS for 30 min at room temperature and protected from light. To assess mitochondrial membrane potential, cells were incubated with 10 μM MitoTracker Red (Invitrogen) in HBSS for 30 min at 37°C in a humidified atmosphere with 5% CO_2_. After staining, cells were washed twice with HBSS or PBS and harvested with 0.25% trypsin-EDTA solution. Following centrifugation at 94*g* for 3 min at 4°C, cell pellets were resuspended in cold 1× PBS with 2% FBS. The intensity of MitoSOX, EthD-1, and MitoTracker Red was measured by flow cytometry using a BD FACSCanto II instrument (BD Biosciences), with excitation at 561 nm and emission detection using a 620/15-nm filter. Data analysis was performed using BD FACSDiva v.6.1.3 software, and three independent experiments were conducted.

### Measurement of mitophagy using mt-Keima fluorescent reporter

A human embryonic kidney (HEK) 293 cell line stably expressing mt-Keima was donated by J. Yun (Dong-A University, Korea) (*53*) and previously described (*54*). Briefly, siRNAs were transfected into HEK293 cells stably expressing mt-Keima. After 48 hours, cells were treated with either dimethyl sulfoxide (DMSO) or 20 μM CCCP for 16 hours in culture medium at 37°C in a humidified atmosphere containing 5% CO_2_. Cells were then harvested using 0.25% trypsin-EDTA and centrifuged at 2500*g* for 3 min at 4°C. The resulting cell pellets were resuspended in cold 1× PBS containing 2% FBS. Mitophagy levels were quantified by flow cytometry. Cells were excited simultaneously with a 405-nm violet laser (emission, 610 ± 10 nm; BV605 detector) and a 561-nm yellow-green laser (emission, 610 ± 10 nm; PE-CF594 detector). A dot plot of BV605 versus PE-CF594 was generated using linear scale settings. Basal mitophagy activity in control cells was categorized as “low,” defined by a PE-CF594/BV605 emission ratio below 15%. Cells exhibiting high mt-Keima fluorescence (PE-CF594/BV605 ratio above 15%) were designated as the “high” mitophagy population. The “percent of parent” (%parent) value indicates the proportion of cells within the mitophagy-induced population. A minimum of 20,000 events were recorded for mt-Keima–positive cells in each sample. Flow cytometry analysis was performed using a BD FACSAria III instrument (BD Biosciences), and the experiments were independently repeated three times. Data were analyzed using BD FACSDiva software v6.1.3.
