# Supplementary material for: In situ characterization of mitochondrial Hsp60-Hsp10 chaperone complex under folding stress
Source: Sci Adv. 2025 Oct 22;11(43):eadw6064. doi: 10.1126/sciadv.adw6064 (PMC12542933; doi:10.1126/sciadv.adw6064)
Supplement: Supplementary file 1 — Figs. S1 to S10 [file sciadv.adw6064_sm.pdf]

Science Advances
AAAS

Supplementary Materials for

In situ characterization of mitochondrial Hsp60-Hsp10 chaperone complex under folding stress

Mingyu Jung *et al.*

Corresponding author: Soung-Hun Roh, shroh@snu.ac.kr

Sci. Adv. **11**, eadw6064 (2025)
DOI: 10.1126/sciadv.adw6064

This PDF file includes:

Figs. S1 to S10

Supplementary figures and legends

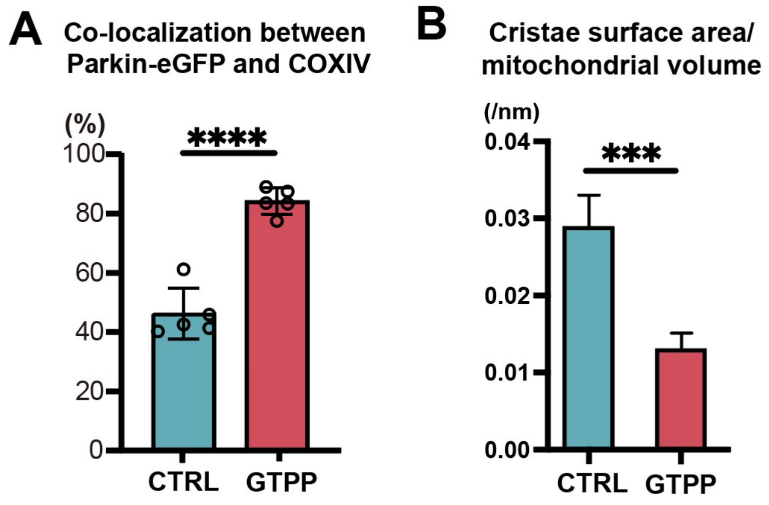

Fig. S1. Mitochondrial co-localization and ultrastructural changes following GTPP treatment. A. Quantification of co-localization between Parkin–eGFP and COXIV in control (CTRL) and GTPP-treated groups (n = 5 biological replicates). **B.** Comparison of cristae surface area normalized to mitochondrial volume between control (n = 18 mitochondria) and GTPP-treated (n = 28 mitochondria) cells. A-B Data are presented as mean ± S.D. (A) or mean ± S.E.M. (B). Statistical significance was determined using unpaired t-tests. ***P < 0.001, ****P < 0.0001.

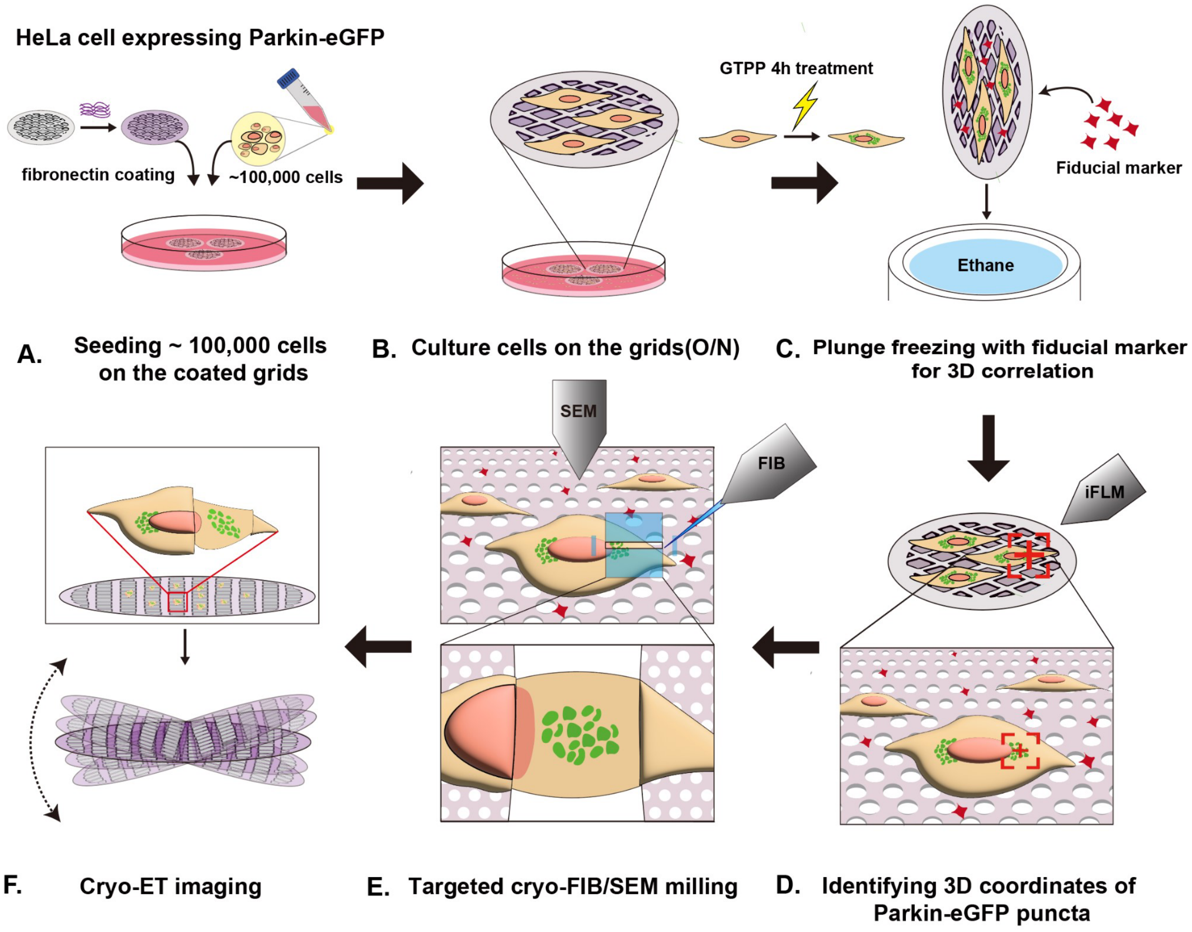

Fig. S2. Schematic workflow of cryo-ET analysis of Parkin–eGFP-expressing HeLa cells under folding stress. A. Fibronectin-coated EM grids are seeded with ~100,000 HeLa cells stably expressing Parkin–eGFP. **B.** Cells are cultured overnight (O/N) for adherence and then treated with GTPP for 4 hours to induce mitochondrial folding stress. **C.** Grids are plunge-frozen in liquid ethane in the presence of fiducial markers to enable 3D correlation. **D.** Cryo-fluorescence light microscopy (iFLM) is used to identify the 3D coordinates of Parkin–eGFP puncta. **E.** Targeted cryo-FIB/SEM milling is performed at the identified coordinates to prepare lamellae. **F.** Cryo-electron tomography (cryo-ET) is conducted to visualize subcellular structures *in situ* at high resolution.

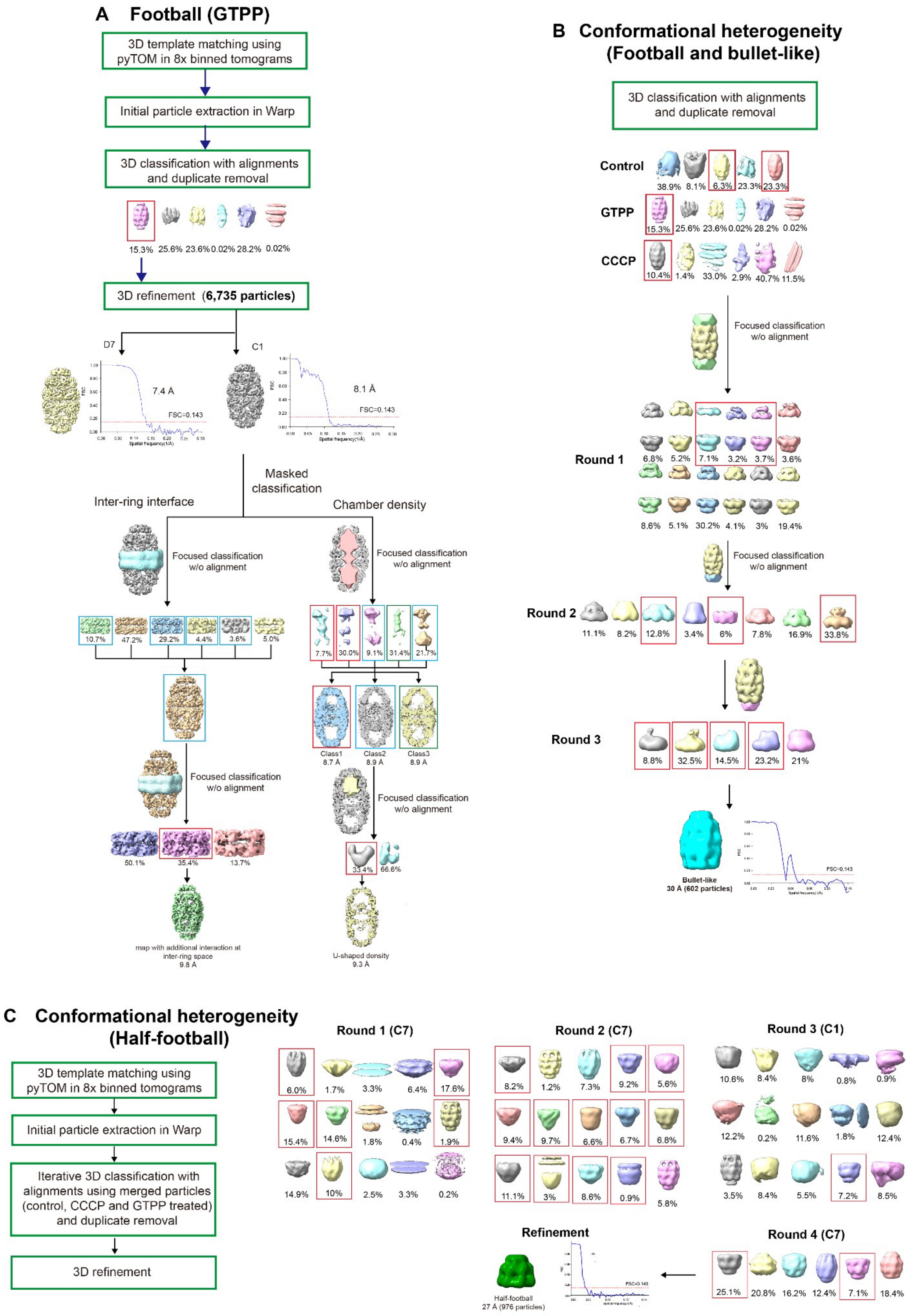
A Football (GTPP)
3D template matching using pyTOM in 8x binned tomograms
Initial particle extraction in Warp
3D classification with alignments and duplicate removal
15.3% 25.6% 23.6% 0.02% 28.2% 0.02%
3D refinement (6,735 particles)
D7
C1
7.4 Å
8.1 Å
FSC=0.143
Masked classification
Inter-ring interface
Chamber density
Focused classification w/o alignment
10.7% 47.2% 29.2% 4.4% 3.6% 5.0%
7.7% 30.0% 9.1% 31.4% 21.7%
Class1 8.7 Å
Class2 8.9 Å
Class3 8.9 Å
50.1% 35.4% 13.7%
33.4% 66.6%
map with additional interaction at inter-ring space 9.8 Å
U-shaped density 9.3 Å
B Conformational heterogeneity (Football and bullet-like)
3D classification with alignments and duplicate removal
Control
38.9% 8.1% 6.3% 23.3% 23.3%
GTPP
15.3% 25.6% 23.6% 0.02% 28.2% 0.02%
CCCP
10.4% 1.4% 33.0% 2.9% 40.7% 11.5%
Round 1
6.8% 5.2% 7.1% 3.2% 3.7% 3.6%
8.6% 5.1% 30.2% 4.1% 3% 19.4%
Round 2
11.1% 8.2% 12.8% 3.4% 6% 7.8% 16.9% 33.8%
Round 3
8.8% 32.5% 14.5% 23.2% 21%
Bullet-like
30 Å (602 particles)
C Conformational heterogeneity (Half-football)
3D template matching using pyTOM in 8x binned tomograms
Initial particle extraction in Warp
Iterative 3D classification with alignments using merged particles (control, CCCP and GTPP treated) and duplicate removal
3D refinement
Round 1 (C7)
6.0% 1.7% 3.3% 6.4% 17.6%
15.4% 14.6% 1.8% 0.4% 1.9%
14.9% 10% 2.5% 3.3% 0.2%
Round 2 (C7)
8.2% 1.2% 7.3% 9.2% 5.6%
9.4% 9.7% 6.6% 6.7% 6.8%
11.1% 3% 8.6% 0.9% 5.8%
Round 3 (C1)
10.6% 8.4% 8% 0.8% 0.9%
12.2% 0.2% 11.6% 1.8% 12.4%
3.5% 8.4% 5.5% 7.2% 8.5%
Round 4 (C7)
25.1% 20.8% 16.2% 12.4% 7.1% 18.4%
Refinement
Half-football
27 Å (976 particles)

Fig. S3. Workflow of cryo-ET subtomogram averaging and classification.

A. Subtomogram averaging workflow for the football-shaped mtHsp60–Hsp10 complex under folding stress (GTPP treatment). **B.** Structural heterogeneity of mtHsp60–Hsp10 complexes under different stress conditions (Control, GTPP, CCCP), as assessed by 3D classification with alignment. A subset of particles was further classified without alignment to resolve the bullet-like conformation. **C.** Subtomogram averaging and classification workflow for the half-football conformation.

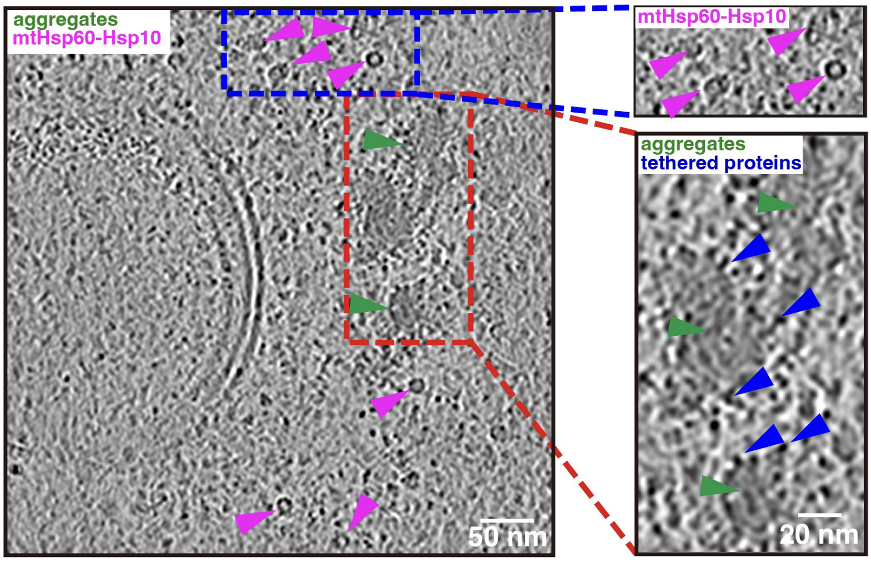

Fig. S4. Representative tomographic slice showing mtHsp60–Hsp10 complexes, aggregates, and tethered proteins in GTPP-treated cells. A tomographic slice from a cryo-electron tomogram of GTPP-treated cells (left) is shown with magnified insets (right). Magenta arrowheads mark mtHsp60–Hsp10 complexes, green arrowheads indicate amorphous protein aggregates, and blue arrowheads denote small tethered protein densities associated with aggregates. Scale bars: 50 nm (left); 20 nm (right).

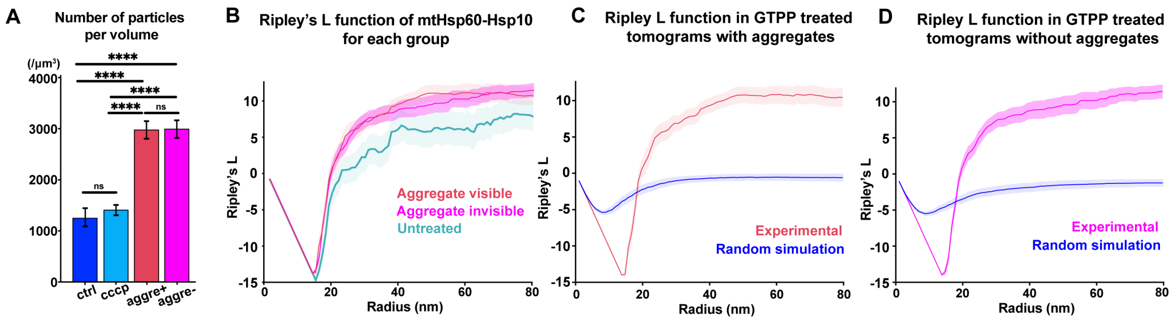

Fig. S5. Concentration and spatial clustering of mtHsp60–Hsp10 complexes assessed by Ripley's L function. A. Comparison of the number of particles among untreated, CCCP treated, aggregates visible and invisible tomograms under folding stress. One-way ANOVA followed by post-hoc Tukey's test was performed to assess statistical significance. ****$P < 0.0001$ **B.** Ripley's L function comparing mtHsp60–Hsp10 spatial distributions in untreated tomograms (cyan), GTPP-treated tomograms with visible aggregates (red), and GTPP-treated tomograms without visible aggregates (magenta). **C.** Ripley's L function for mtHsp60–Hsp10 in GTPP-treated tomograms with aggregates (n = 32). **D.** Ripley's L function in GTPP-treated tomograms without aggregates (n = 113). In all panels, bold lines represent mean values and shaded areas indicate mean ± S.E.M. Blue lines represent random simulation controls. A shift above the random line indicates significant spatial clustering.

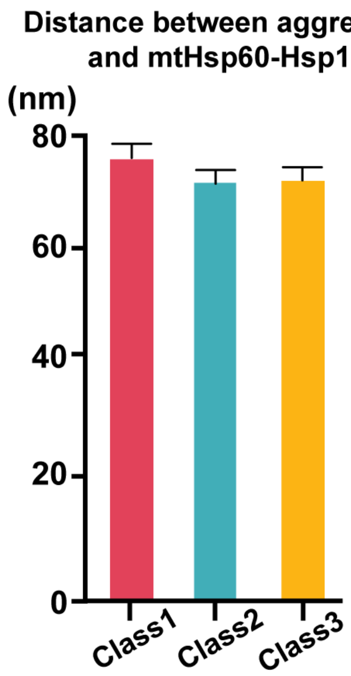

Fig. S6. Quantification of distances between aggregates and mtHsp60–Hsp10 complexes across structural classes. Bar graph showing the average distance between aggregates and mtHsp60–Hsp10 complexes for Class 1, Class 2, and Class 3 tomographic reconstructions. Data are presented as mean ± S.D. No statistically significant differences were observed among the classes.

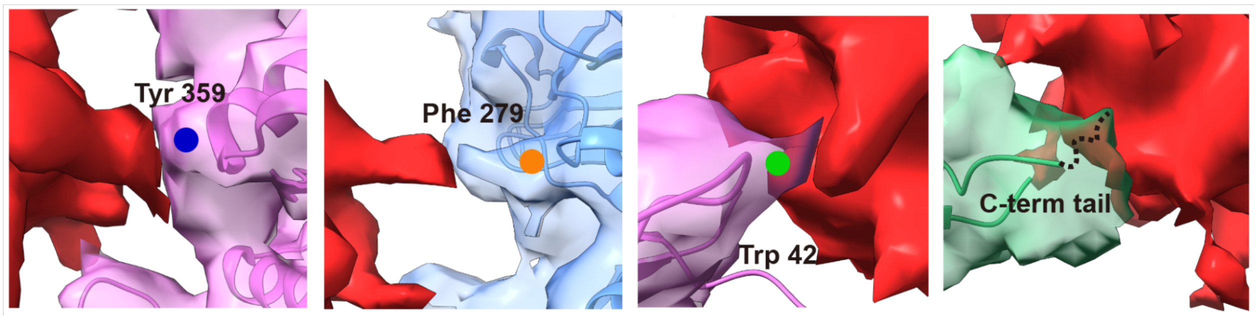

Fig. S7. Four distinct substrate contact sites within the mtHsp60 chamber. Close-up views of four representative interaction sites between mtHsp60 and the bound substrate density (red). Key contact residues are labeled and highlighted: Tyr359 (blue) and Phe279 (orange) in the apical domain, Trp42 (green) in the equatorial domain, and the C-terminal tail (brown) extending from the equatorial domain.

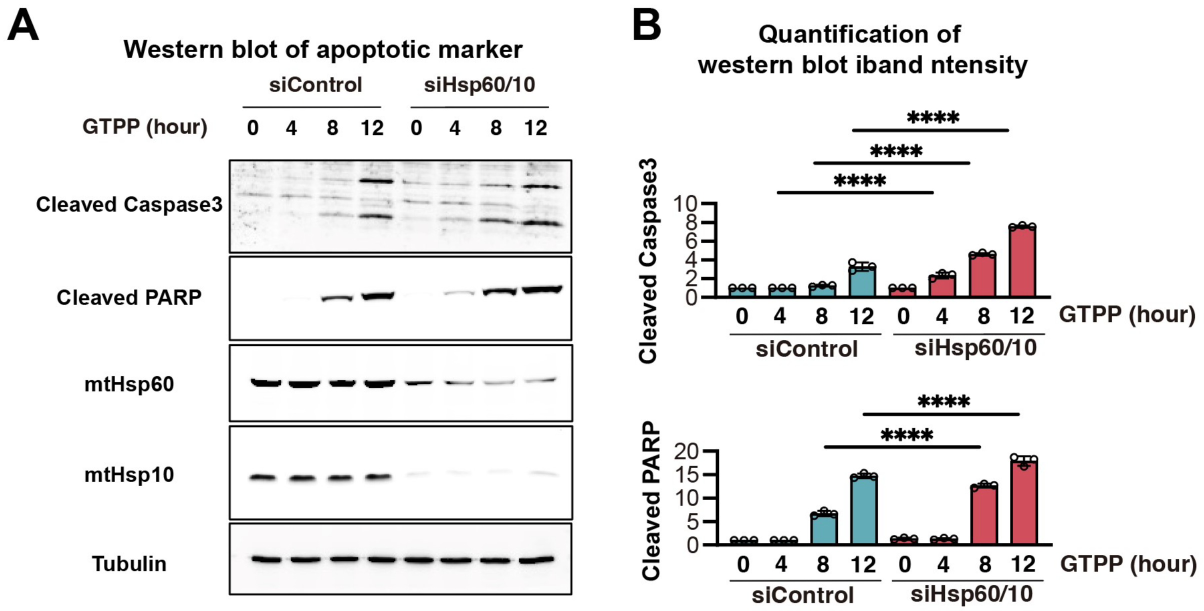

Fig. S8. Apoptotic responses upon mtHsp60–Hsp10 knockdown under folding stress. A. Western blot analysis of apoptotic markers in siControl and siHsp60/10 cells following GTPP treatment for 0, 4, 8, or 12 hours. Cleaved Caspase-3 and cleaved PARP were used as markers of apoptosis. mtHsp60, mtHsp10, and tubulin were used as controls. **B.** Quantification of band intensities for cleaved Caspase-3 (top) and cleaved PARP (bottom). Data are presented as mean ± S.D. from biological replicates. Statistical significance was assessed using one-way ANOVA followed by Tukey's post hoc test. ****P < 0.0001.

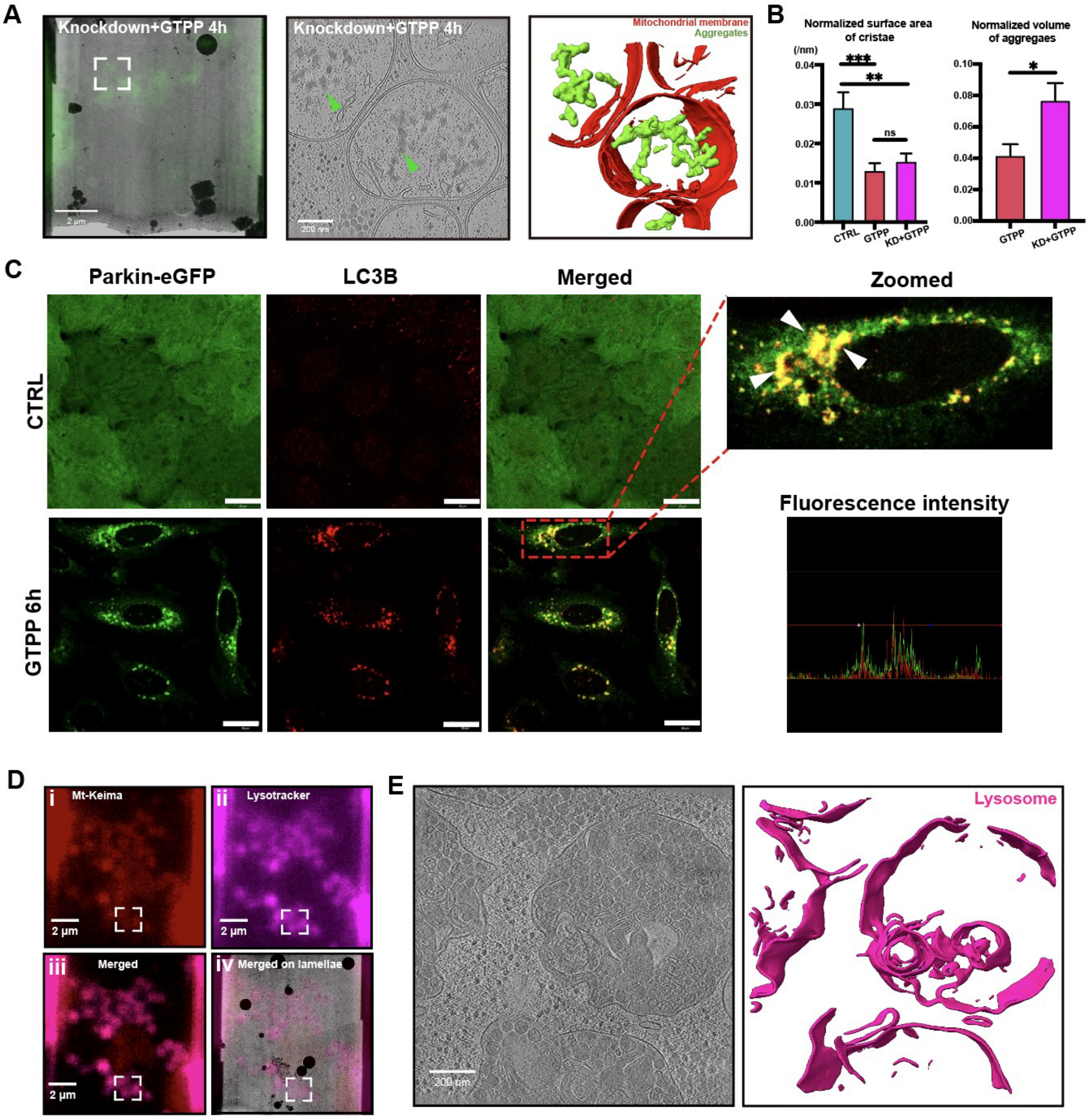

Fig. S9. Mitochondrial remodeling and mitophagy in mtHsp60–Hsp10 knockdown cells under folding stress. A. cryo-ET of mtHsp60–Hsp10 knockdown cells treated with GTPP for 4 h. Left: aligned cryo-fluorescence image overlaid on lamella montage; middle: representative tomographic slice showing aggregates (green arrowheads); right: segmented 3D reconstruction showing mitochondrial membranes (red) and aggregates (green). **B.** Quantification of mitochondrial architecture. Left: normalized cristae surface area; right: normalized aggregate volume in GTPP-treated wild-type and knockdown cells. Mitochondria analyzed: CTRL (n = 18 mitochondria), GTPP (n = 28 mitochondria), KD + GTPP (n = 46 mitochondria). Data are presented as mean ± S.E.M. Statistical analysis was performed using one-way ANOVA with Tukey's post hoc test and unpaired t-tests. **C.** Immunofluorescence images of Parkin–eGFP (green) and LC3B (red) under control or GTPP-treated (6 h) conditions. Zoomed inset highlights co-localized puncta (arrowheads), and fluorescence intensity profile confirms signal overlap. **D.** Cryo-CLEM imaging using mt-Keima (red) and LysoTracker (magenta) to target mitochondria undergoing lysosomal degradation. Co-localized signals were aligned to lamella montages to guide downstream cryo-ET. **E.** Representative tomographic slice (left) and

corresponding segmented 3D model (right) showing lysosomes (magenta) in the targeted region.

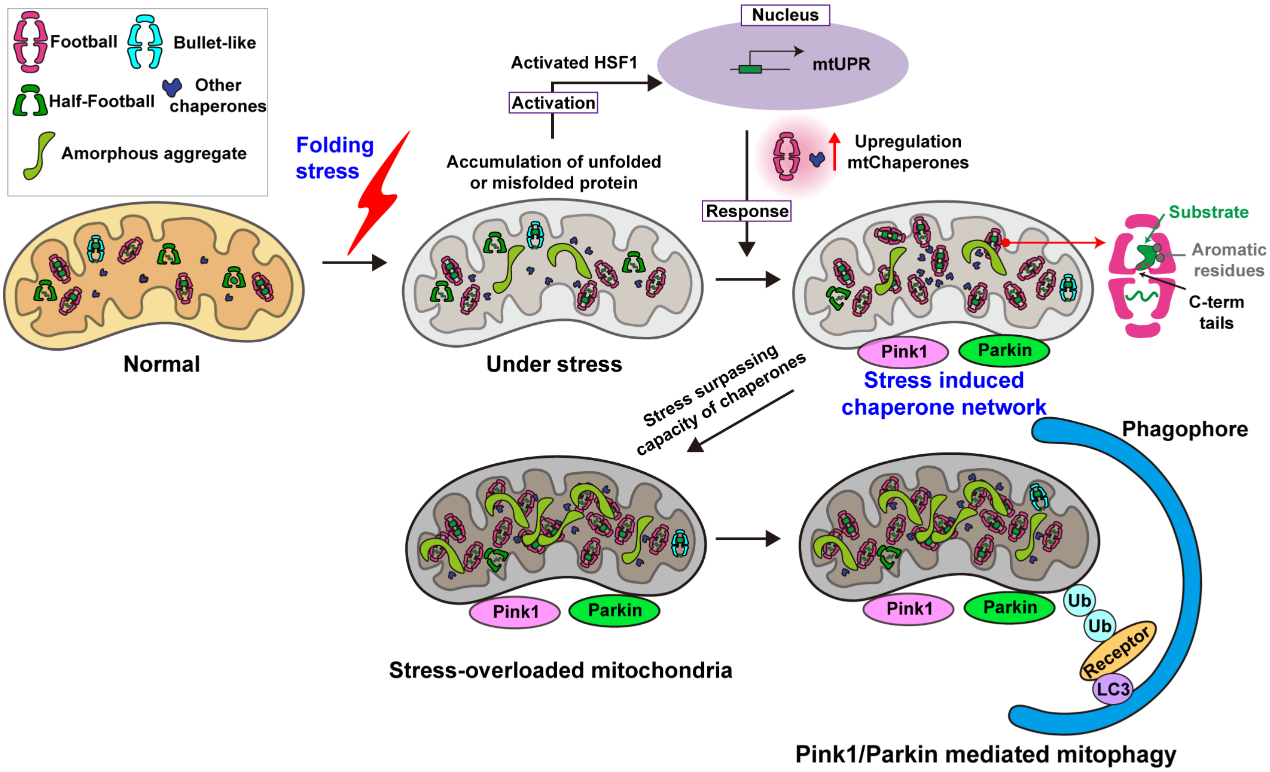

Fig. S10. Proposed mechanism and molecular networks of mtUPR. When normal mitochondria experience folding stress, unfolded proteins accumulate and form amorphous aggregates. This disrupts protein import, leading to activation of HSF1. Activated HSF1 then expresses genes encoding mitochondrial chaperones, including mtHsp60-Hsp10. The upregulated chaperones are transported into the mitochondria. In the mitochondria, smaller chaperones tether to and disaggregate the aggregates. The mtHsp60-Hsp10 complexes subsequently encapsulate the disaggregated products from the smaller chaperones. These coordinated chaperone networks work together to actively mitigate the folding stress. Stress-overloaded mitochondria, despite the active work of chaperones, were selectively degraded through PINK1/Parkin-mediated mitophagy to preserve cellular homeostasis.
